# A Prospective Observational Study on the Outcome Assessment of Conservative Management Versus Intercostal Drainage (ICD) in Blunt Chest Injury Patients With ≤3 Rib Fractures in a North Indian Tertiary Care Center

**DOI:** 10.7759/cureus.42167

**Published:** 2023-07-19

**Authors:** Shreedhar Shandilya, Shubhajeet Roy, Anurag Rai, Suresh Kumar, Shailendra Kumar, Sandeep Tiwari, Abhinav A Sonkar

**Affiliations:** 1 General Surgery, King George's Medical University, Lucknow, IND; 2 Medical Sciences, King George's Medical University, Lucknow, IND; 3 Thoracic Surgery, King George's Medical University, Lucknow, IND; 4 Trauma Surgery, King George's Medical University, Lucknow, IND

**Keywords:** trauma, intercostal drainage, conservative, rib fracture, ttss, blunt chest injury

## Abstract

Introduction

Trauma is the third most common cause of death in all age groups. One out of four trauma patients die due to thoracic injury or its complications. Seventy percent of thoracic traumas are due to blunt injury. This indicates the importance of chest trauma among all traumas. Quick and precise assessment bears paramount importance in deciding life-saving and definitive management. Often, the initial management in blunt injury patients is based on subjective assessment by the attending clinician. A scoring system that provides early identification of the patients at the greatest risk for respiratory failure and more likely to require mechanical ventilation and require prolonged care, as well as those with a higher mortality risk, may allow the early institution of intervention to improve outcomes. Thoracic Trauma Severity Score (TTSS) poses to be a precise tool in directing the management modality to be employed.

Methodology

This was an observational study including 112 patients of age >12 years, with blunt chest injury, sustaining ≤3 rib fractures, and with a stable chest wall. The patients with penetrating injury, those with blunt chest injury having flail segment, patients in the pediatric age group (<12 years), or polytrauma patients were excluded from our study. Of the 112 patients, 56 had been managed by intercostal drainage (ICD), and the rest (56) had been managed conservatively.

Result

Road traffic accidents (RTA) were the most common mode of injury in both groups. The percentage of the patients with one, two, and three rib fractures was 57.14%, 32.14%, and 10.71%, respectively, in the ICD group and 85.71%, 7.14%, and 7.14%, respectively, in the conservative management group (p = 0.124). The mean TTSS score was significantly more in the ICD group as compared to the conservative management group in the single rib fracture patients (p = 0.001*), as well as all patients of any number of rib fractures (p < 0.01*) (significance was defined as a value of p less than 0.05 {indicated by an asterisk}). The mean hospital stay was significantly lower in the conservative group as compared to the ICD group (p < 0.01*). The mean SF-36 (outcome) was significantly more in the conservative management group as compared to the ICD group (p = 0.020*). The mean cost of treatment was significantly more in the ICD group as compared to the conservative management group (p < 0.001*).

Conclusion

In our study, a TTSS (as measured by the primary care surgeon) of >7, across any number of rib fractures, was preferably predictive of management by ICD, while a <7 value was favorable for conservative management. TTSS can be used as an important tool to predict the management modality in blunt chest injury patients with ≤3 rib fractures.

## Introduction

Trauma is the third most common cause of death worldwide, in all age groups. One out of four trauma patients die due to thoracic injury or its complications. Seventy percent of thoracic traumas are due to blunt chest injuries [1,2]. This indicates the importance of chest trauma over all other traumas. In blunt trauma, all the structures within the thorax can be damaged, such as the chest wall soft tissues, thoracic cage, ribs, lungs, pleura, large vessels, diaphragm, heart, and mediastinal structures [3]. Multiple rib fractures, resulting in a flail chest, affect respiratory mechanics and cause increased work of breathing, with associated pulmonary contusion spanning multiple lung lobes [4]. Chest injury patients may also sustain pulmonary contusion and lacerations. Such injuries take 48-72 hours to evolve.

Hemothorax is an accumulation of blood within the pleural cavity, primarily caused by lung contusion or laceration and the involvement of an intercostal vessel, the great vessels, or the internal mammary artery. The bleeding, often a self-limited phenomenon, seldom needs operative interventions [5]. Some of the chest injuries leading to hemothorax can be managed conservatively without chest tube drainage, provided it is mild or small, and the patient can maintain oxygenation with oxygen supplementation and adequate analgesia. Patients with retained hemothorax or massive hemothorax with a flow of more than 1500 mL of blood in the chest drain may urgently require thoracotomy to explore the source of bleeding. The patients, albeit with an initial output of less than 1500 mL of fluid but with continuous drainage of blood (200 mL/hour for 2-4 hours), may also require urgent thoracotomy. Also, a hemodynamically unstable patient, with repeated blood transfusion requirement, equally warrants the need for emergency thoracotomy to explore the cause [6,7].

Erect chest X-ray, a readily accessible, quick investigation, although failing to exhibit the extent of injuries, allows the early identification of life-threatening injuries such as massive hemothorax, pneumothorax, or any fracture of the ribs. Quick and precise assessment bears paramount importance in deciding life-saving and definitive management. The initial management is performed as per the Advanced Trauma Life Support (ATLS) guidelines. A scoring system that provides the early identification of the patients at the greatest risk for respiratory failure and more likely to require mechanical ventilation and prolonged care, as well as those with a higher mortality risk, may allow the early institution of intervention to improve outcomes [8]. Pape et al. described the Thoracic Trauma Severity Score (TTSS), a scale that included both anatomical and functional parameters. The purpose of the scale was to help emergency medical evaluation in identifying trauma patients at risk of pulmonary complications, using parameters available during the initial evaluation, which could be applied in primary- and secondary-level hospitals [9]. The TTSS is an appropriate and feasible tool to predict the development of complications or mortality mostly in patients with mild thoracic trauma [10,11].

Lema et al. (2011) conducted a prospective study on 150 chest trauma patients. Males dominated the tally over females by a ratio of 3.8:1. Of the patients, 55.3% were managed by nonoperative methods, whereas 19.3% of the patients required underwater seal drainage. The mean hospital length of stay (LOS) was 13.17 days, and the rate of mortality was 3.3% [12]. Kesieme et al. (2011) did a literature review on tube thoracostomy (TT) done from 1970 to April 2011. The review study updated on the complications and the management of complications encountered after tube thoracostomy. Following a tube thoracostomy, the most common infectious complications include empyema and surgical site infection (SSI). Though performed commonly, TT is not a procedure without risks [13]. Pressley et al. (2012) conducted a trauma registry review on the patients with rib fracture from June 2008 to February 2010 at level I trauma center, in which a simple scoring system was developed to stratify the risk of respiratory failure and was applied to patients retrospectively. The scoring system was applied to 649 patients in a retrospective fashion to determine its validity. Chest wall trauma scoring system predicts the likelihood of the need for mechanical ventilation and prolonged hospital care. A score of 7 or 8 predicted increased mortality risk, intensive care unit (ICU) admission, and intubation. A score of >5 predicted a longer LOS and longer ICU admission. The scoring system could assist in the earlier optimization of treatment plan such epidural anesthesia, operative fixation of fractures, and postoperative ICU support and care [10].

Goodman et al. (2013) did a query on trauma registry at level I trauma center from 1999 to 2010 for video-assisted thoracoscopic surgery (VATS) procedure within 24 hours of admission. Thirteen percent of the patients sustained blunt injury, while 87% had penetrating trauma. Indications for urgent VATS included esophageal/diaphragmatic injury, ongoing hemorrhage, retained hemothorax, and open or persistent pneumothorax. The mean postoperative duration of intercostal drainage (ICD) placement was 2.9 days, and the mean LOS was 5.6 days. VATS can be safely employed in the effective management of thoracic trauma in hemodynamically stable patients presenting within 24 hours of injury [14]. Oikonomou and Prassopoulos (2011) conducted a prospective, observational study from December 2009 to January 2012 on the patients >14 years of age with blunt chest trauma at nine US level I trauma centers. The study aimed to determine the diagnostic yield of blunt chest trauma imaging: chest X-ray and chest computed tomography (CT) scan. Chest injury as seen on chest imaging and its clinical significance were defined by a trauma expert panel. The yield of imaging was calculated as follows: chest X-ray, 8.4 ± 0.5%; chest CT, 28.8 ± 1.8%; and chest CT after normal chest X-ray, 15.0 ± 1.2% [15].

Unsworth et al. (2015) did a literature review across 977 articles published from 1990 to March 2014 to analyze interventions done in rib fracture and consequent impact on the patient and their outcome. Blunt chest trauma patients develop complications secondary to rib fractures on the account of pain and inadequate ventilation. Clinical protocols, analgesic management, surgical fixation, and other treatments were the categories under which the clinical interventions were categorized. In the patients with flail chest, surgical fixation significantly improved patient outcomes. In the patients with three or more rib fractures, epidural analgesia was superior to both patient-controlled analgesia (PCA) and intravenous narcotics in terms of hospital and patient outcomes, including pain alleviation and pulmonary function. The majority of the publications that were evaluated suggested a multidisciplinary strategy that included nursing, medical, allied health (chest physiotherapy and dietician input), and surgical intervention (the stabilization of flail chest) [16]. Kong et al. (2015) conducted a retrospective study on thoracic trauma patients over a four-year period in a single institution to determine the validity of the policy of selective conservatism. The indications for TT were pneumothorax (38%), hemothorax (30%), and hemopneumothorax (32%). Following tube removal, 13% of all chest X-rays showed persistent abnormalities that prevented prompt discharge: 8% in pneumothorax, 15% in hemothorax, and 16% in hemopneumothorax. No additional treatment was necessary for any of the 32 patients in pneumothorax. For the drainage of residual hemothorax, 17 patients needed repeat TTs, and 27 needed VATS. In hemopneumothorax, 22 patients required VATS, whereas 29 patients needed repeat TTs. TT alone can be used to manage the vast majority of patients with thoracic trauma [17].

Pramod et al. (2014) conducted a retrospective study on 60 patients with traumatic chest injury. Of these, 58.3% of the patients were initially managed without chest tube. Three patients subsequently required chest tube insertion due to the radiological evidence of pneumothorax. During conservative management, none of the patients' conditions got worse. Chest tube insertion may be avoided for mild or small-sized traumatic chest injury, if there are no associated significant injuries [18]. Martínez et al. (2016) conducted a study on thoracic trauma patients (n = 238), middle-aged (62.2 ± 15 years), with falls and traffic accidents as the main mechanism of injury. According to the study, TTSS was significant for predicting complications (area under the curve {AUC} = 0.848) and mortality (AUC = 0.856), with a cutoff value of eight points having sensitivity and specificity of 66% and 94% for predicting complications and 80% and 94% for predicting mortality, respectively. The study concluded that TTSS is an appropriate and feasible tool to predict the development of complications or mortality in mild trauma to the thorax [11].

Huang et al. (2016) conducted a study on 78 patients aged >65 years with blunt injury to the chest and stable vitals who underwent direct VATS without a TT as against those managed by initial TT. All of them had hemothorax of >300 mL, indicating the need for TT. The patients who did not receive a thoracostomy had lower post-trauma infection rates (28.6% versus 56.3%) and a significantly shorter length of ICU stay (3.13 versus 8.27) and overall LOS (15.93 versus 23.17) compared with those who received a thoracostomy [19]. Khursheed et al. (2019) conducted a retrospective analysis of the patients with chest injuries. There were 1429 trauma patients overall who came to the emergency department, and 160 of them (11.2%) experienced chest trauma. The majority of the patients (51.87%) were male and in the age range of 21-40. While gunshot injuries were the main cause of penetrating chest injuries, road traffic accidents (RTA) were the main source of blunt chest injuries. Most frequently, TT was used to treat patients (65%), and conservative treatment was used to treat 28% of the patients [20]. Sikander et al. (2020) found that hemothorax and hemopneumothorax were the most frequent primary presentations, with rib fracture encountered in 90% of the patients. The rate of mortality was 21.3%. Factors significantly related to mortality were age of ≥80 years, tension pneumothorax, preexisting cardiopulmonary disease, blood loss of ≥500 mL, flail chest, and chest trauma score of ≥5. The mean LOS was 5.3 ± 3.4 days. Factors lengthening hospital stay by more than five days included lung contusion, more than two rib fractures, hemopneumothorax, pneumonia, acute respiratory distress syndrome (ARDS), and flail chest. Elderly patients with blunt thoracic trauma had higher mortality [21].

Although many studies have been conducted to investigate the factors that predict morbidity and death in thoracic trauma, only a few have compared the quality of life and the cost of treatment across different modalities, that too in the Indian context. Also, there are very few studies available in the literature that correlate treatment modality outcome with TTSS score, which is a newer and a better tool than its predecessors.

## Materials and methods

An observational study was carried out on 112 patients of age >12 years, with blunt chest injury, sustaining ≤3 rib fractures, and with stable chest wall. The study was conducted for 18 months from February 1, 2021, to July 31, 2022. The study was conducted on the patients, fulfilling the above inclusion criteria, admitted in the departments of general surgery, trauma surgery, and thoracic surgery of a tertiary care university hospital in North India, after taking written informed consent from the patients or their attendants. The patients with penetrating injury, those with blunt chest injury having flail segment, patients in the pediatric age group (<12 years), patients with pneumothorax, or polytrauma patients were excluded from our study. Of the 112 patients, 56 had been managed by intercostal drainage (ICD), and the rest (56) had been managed conservatively (conservative management means management by means of analgesics, oxygen support, and chest physiotherapy). Analgesic treatment included nonsteroidal anti-inflammatory drugs (NSAIDs), epidural catheters, intravenous narcotics, patient-controlled analgesia (PCA), lidocaine patches, intercostal blocks, and paravertebral blocks.

After the primary survey and the stabilization of the patients, a detailed history was elicited, and routine laboratory values were obtained. At presentation, the patient's vital parameters were recorded and optimized, and laboratory investigations such as arterial blood gas (ABG) and routine blood counts were done. Chest X-ray in the anteroposterior (AP) view and contrast-enhanced computed tomography (CECT) of the thorax were also done after the stabilization of the patient (a direct sign of a rib fracture on an axial CT scan of the chest is the separation of two rib fragments with the associated sharp edges). The secondary findings related to rib fractures include a hemothorax, pneumothorax, and lung contusion. These findings are more easily seen on chest CT scans than on chest radiographs. Also, three-dimensional (3D) reconstruction could be done in CT, in doubtful cases. The new Berlin definition of polytrauma was used: a patient with an Abbreviated Injury Scale (AIS) score of ≥3 for two or more different body regions with additional one or more variables from the five physiological parameters, including systolic blood pressure (SBP) of ≤90 mm Hg, Glasgow Coma Scale (GCS) score of ≤8, base excess of ≤6.0, international normalized ratio of ≥1.4 or partial thromboplastin time of ≥40 seconds, and age of ≥70 years [22]. In simpler words, polytrauma comprises more than one system involvement, such as blunt trauma to the chest and abdomen, head and spine injury, musculoskeletal injury, or all combined or in different combinations. The findings of hemothorax, the number of ribs fractured, and the lung contusion were assessed by taking into account the number of lobes involved and the laterality of injury in CECT of the thorax. Thoracic Trauma Severity Score (TTSS) [9] was then calculated for every patient (Table 1).

**Table 1:** Thoracic Trauma Severity Score (TTSS) Source: [9]. PaO_2_, partial pressure of oxygen; FiO_2_, fraction of inspired oxygen

Grade	PaO_2_/FiO_2_	Rib fractures	Lung contusion	Pleura	Age (in years)	Points
0	>400	0	No	No	<30	0
I	300-400	1-3	Unilobar unilateral	Pneumothorax	30-41	1
II	200-300	3-6	Unilobar bilateral or bilobar unilateral	Hemothorax or hemothorax/pneumothorax unilateral	42-54	2
III	150-200	>3 bilateral	Bilateral, <2 lobes	Hemothorax or hemothorax/pneumothorax bilateral	55-70	3
IV	<150	Flail chest	Bilateral, ≥2 lobes	Tension pneumothorax	>70	5

The patients were kept under observation, catering to their analgesic requirements, and chest physiotherapy with pulmonary toileting was done. Chest X-ray in the AP view was repeated on day 7, day 14, and month 1 (day 30) follow-up to look for complications. The patients were sent for ultrasonography (USG) of the thorax on day 7 of admission and at one-month follow-up to look for any evidence of residual collections (hemothorax). The factors analyzed were demographic details, mode of injury, associated injuries, number of rib fractures, comorbidities, management modality, Thoracic Trauma Severity Score (TTSS), complications, hospital length of stay (LOS), quality of life in terms of SF-36 questionnaire [22] at one-month follow-up, and cost of treatment.

The study was conducted after being approved by the Institutional Ethics Committee of King George's Medical University (reference number: V-PGTSC-IIA/P16). The statistical analysis was done using the Statistical Package for Social Sciences (SPSS) version 23.0 (IBM SPSS Statistics, Armonk, NY). Tests of significance such as the chi-square test and Z-test were used whenever necessary. Significance was defined as a value of p less than 0.05 (indicated by an asterisk {*}).

## Results

A total of 112 patients were enrolled in the study, in which 56 (50.0%) of the patients in the ICD group and 56 (50%) of the patients in the conservative management group were included. Males comprised 88 (78.57%) of the study population and females 24 (21.42%). The number of males and females was 48 (85.71%) and eight (14.29%) in the ICD group and 40 (71.43%) and 16 (28.57%) in the conservative management group, respectively. Based on gender, both groups were comparable (p = 0.383). The mean age was 38.77 ± 12.60 years in the ICD group and 41.93 ± 12.03 years in the conservative management group. The mean age was not significantly different between groups (p = 0.400). The modes of blunt injury to the chest have been summarized in Table 2, with RTA being the most common of them all.

**Table 2:** Comparison of the modes of injury in the patients undergoing different management modalities for blunt chest injury. ICD: intercostal drainage

Modes of blunt chest injury	Total number of patients	Total percentage	ICD group		Conservative management group		Chi-square	p-value
			Number of patients	Percentage	Number of patients	Percentage		
Road traffic accident (RTA)	80	71.42%	36	64.28%	44	78.57%	1.97	5.78
Fall from height	26	23.21%	14	25.00%	12	21.43%		
Assault	3	2.68%	3	5.36%	0	0.00		
Bull horn injury	3	2.68%	3	5.36%	0	0.00		

The percentage of the patients with one, two, and three rib fractures were 57.14%, 32.14%, and 10.71%, respectively, in the ICD group and 85.71%, 7.14%, and 7.14%, respectively, in the conservative management group (p = 0.124).

The TTSS was compared between the ICD group and the conservative management group, against the number of rib fractures (Table 3). The mean TTSS score was significantly more in the ICD group as compared to the conservative management group in the single rib fracture patients (p = 0.001*), whereas in two and three rib fractures, the mean TTSS score was insignificantly more in the ICD group as compared to the conservative management group. The overall mean TTSS was significantly more in the ICD group as compared to the conservative management group (p < 0.01*). The lower limit of TTSS for the ICD group was 7.11, while the higher limit for the conservative group was 7.98.

**Table 3:** Comparison of mean TTSS in different management modalities for blunt chest injury against the number of ribs fractured. *P-value is significant (<0.05). ICD, intercostal drainage; TTSS, Thoracic Trauma Severity Score

	ICD group (n = 56)				Conservative management group (n = 56)				t score	p-value
Number of ribs fractured	Number of patients	Percentage	Mean TTSS	±SD	Number of patients	Percentage	Mean TTSS	±SD		
1	32	57.14%	8.31	1.33	48	85.71%	6.58	1.31	3.85	0.001*
2	18	32.14%	8.39	1.09	4	7.14%	7.00	0.00	1.24	0.233
3	6	10.71%	9.67	1.97	4	7.14%	8.00	0.00	0.79	0.468
All patients	56	100.00%	8.48	1.37	56	100.00%	6.71	1.27	4.37	<0.001*

The mean hospital length of stay was 9.91 ± 4.67 days in the ICD group and 4.79 ± 3.29 days in the conservative management group. The mean hospital stay was significantly lower in the conservative group as compared to the ICD group (p < 0.01*).

Lung complication at day 0, day 7, day 14, and day 30 in different management modalities for blunt chest injury has been shown in Table 4.

**Table 4:** Lung complication at day 0, day 7, day 14, and day 30 in different management modalities for blunt chest injury ICD: intercostal drainage

Day	Lung complications	ICD group		Conservative management group		Chi-square	p-value
		Number of patients	Percentage	Number of patients	Percentage		
Day 0		43	76.78%	36	64.28%	0.33	0.567
	Atelectasis	6	10.71%	4	7.14%		
	Fever	13	23.21%	12	21.43%		
	Fever and atelectasis	24	42.86%	20	35.71%		
Day 7	Atelectasis	30	53.57%	24	42.85%	2.43	0.119
Day 14		10	17.85%	0	0.00%	1.35	0.246
	Atelectasis	6	10.71%	0	0.00%		
	Empyema	4	7.14%	1	1.79%		
Day 30	Empyema	4	7.14%	0	0.00%	1.95	0.163

The mean SF-36 (outcome) was 62.39 ± 7.12 in the ICD group and 67.00 ± 2.32 in the conservative management group. The mean SF-36 (outcome) was significantly more in the conservative management group as compared to the ICD group (p = 0.020*).

The mean cost of treatment was ₹14625.00 ± ₹3763.76 in the ICD group and ₹8000.00 ± ₹2385.86 in the conservative management group. The mean cost of treatment was significantly more in the ICD group, as compared to the conservative management group (p < 0.001*).

## Discussion

Chest trauma is one of the most common injuries suffered by trauma patients and is also the second leading cause of physical trauma-related death, only secondary to head injury. About one-fourth of all trauma-related deaths are found to have a primary or contributing role in chest injury [17]. Due to an increase in traffic accidents (6% of all traffic accidents worldwide), the availability of modern high-speed automobiles, people's inexperience, and the lack of awareness of traffic laws, chest trauma is a prominent cause of accidental deaths in India [23,15]. Thoracic injuries may be treated by conservative methods or by invasive interventions [1]. The thoracic viscera and the chest wall are both impacted by chest injuries. A blend of these wounds is frequently found. As the thoracic cage overlaps the abdomen, damage to other organs, particularly the abdominal solid organs, is frequently linked to thoracic trauma. The management of chest injuries should be done with the utmost urgency because they tend to be the primary reasons behind deaths. The management of critically ill patients has significantly improved owing to the use of blood transfusion, artificial ventilators, antibiotics, and more accurate diagnostic modalities. The management of chest injuries has taken leaps, with the onus resting on better physiotherapy and rehabilitation services. The number of preventable deaths following trauma has been noted to decrease with the advent of the use of sophisticated prehospital and trauma care protocols; nonetheless, preventative strategies must have made the greatest influence on lowering the burden of trauma [24,25]. Inserting an intercostal chest drain is a pertinent skill in the care of trauma patients. However, there is a 30% possibility of complications after the procedure, with numerous case reports describing instances of deadly aftermaths of ICD insertion, such as ventricular perforation and pulmonary artery damage [16].

In our study, the mean age was 38.77 ± 12.60 years in the ICD group and 41.93 ± 12.03 years in the conservative management group (p = 0.400). The mean age was not significantly different between the two groups. Yimam et al. (2021) reported that the average age of chest injury patients was 36.17 ± 15.6 years [26]. Veysi et al. (2009) observed that when chest trauma occurs, the average age is 40.8 years (median, 37; range, 16-100) [27]. According to Harde et al. (2019), the patients treated with isolated chest trauma had an average age of 34.50 ± 15.861 years [28]. The average age, according to Huber et al. (2014), was 46.1 years [29].

In our study, the percentage of males and females was 85.71% and 14.29%, respectively, in the ICD group and 71.43% and 28.57%, respectively, in the conservative management group (p = 0.383). The overall male/female ratio was 3.67. Yimam et al. (2021) [26] reported that nearly three-fourths (76.6%) of chest trauma patients were males. The male/female ratio was 3:1. Males were found to be the most vulnerable group to RTA-related deaths, as per Singh (2017) [30].

In our study, the percentage of falls from height, RTA, assault, and bull horn injury was 25.00%, 62.50%, 5.36%, and 5.36%, respectively, in the ICD group and 21.43%, 78.57%, 0.00%, and 0.00%, respectively, in the conservative management group (p = 5.78). According to Hajjar et al. (2021), RTA was found to be the most frequent cause of chest injury (n = 205, 86.9%) [31]. According to Kong et al. (2015), 55% (136/248) of the 248 patients who suffered blunt trauma did so as a result of a motor vehicle accident (RTA), 39% (97/248) as a result of an assault, and 6% (15/248) as a result of a fall [17].

In our study, the mean length of stay (LOS) was 9.91 ± 4.67 days in the ICD group and 4.79 ± 3.29 days in the conservative management group (p < 0.01*). According to Hajjar et al. (2021), 183 (77.5%) blunt chest injury patients spent longer than three days in the hospital [31]. The average LOS for the patients receiving conservative management was shorter than that for patients receiving tube thoracostomy [31-35]. Pressley et al. (2012) conducted a study through a retrospective trauma registry review in which a simple scoring system was employed to predict the likelihood of ventilator requirement and prolonged LOS. The increased risk of admission to the ICU, intubation, and mortality was signified by a score of 7 or 8, whereas a score of >5 was favorable for a longer LOS and a longer ventilator requirement [10]. In our study, the mean SF-36 (outcome) was 62.39 ± 7.12 in the ICD group and 67.00 ± 2.32 in the conservative management group. The mean SF-36 (outcome) was significantly more in the conservative group as compared to the ICD group, testifying the quality of life to be better in the conservative management group as compared to the ICD group.

In our study, the percentage of lung complications was 69.64%, 41.07%, 16.07%, and 19.65% in the ICD group and 57.14%, 14.29%, 0.00%, and 0.00% in the conservative management group at day 0, day 7, day 14, and day 30, respectively. The lung complication was significantly more in the ICD group as compared to the conservative management group. In our study, only one patient, who was managed by conservative methods, developed empyema at day 14 follow-up visit, and thereby, tube thoracostomy was done for its management. In a study by Puri et al. (2021), the overall complication rate was 3.6% (four instances), with two occurrences of pleural collection, one of which needed the chest drain to be reinserted and the other was treated conservatively [36]. None of the patients enrolled on our study for observation developed retained hemothorax, as confirmed by USG of the thorax on day 7 and day 30 follow-up. One patient, initially managed by the conservative method, developed empyema, evident from day 14 follow-up X-ray, and was further managed by tube thoracostomy.

The mean TTSS score was significantly higher in the ICD group as compared to the conservative management group in one rib fracture, as well as in all patients of any number of rib fractures. To aid in the early identification of the injury, there should be a scale, which is precise, reliable, and specific. Looking at the impact of thoracic injury, the scale should take into account anatomical and physiological parameters. The validation of a scale needs to be implemented on a population different from the one it has been described on. TTSS is a specific scale for thoracic trauma, originally conceived for the patients with severe blunt trauma (AIS > 2; ISS > 18) but with more than 48 hours of survival [11]. In a study, Pressley et al. (2012) conducted a trauma registry review of the patients with rib fractures at a level I trauma center on 649 patients, in which a simple scoring system was developed to stratify the risk of respiratory failure and was applied to patients retrospectively to determine its validity [10]. Chest wall trauma scoring system predicts the likelihood of the need for mechanical ventilation and prolonged hospital care. A score of 7 or 8 predicted increased mortality risk, ICU admission, and intubation. A score of >5 predicted a longer LOS and longer ICU admission [10]. Martínez et al. (2016) concluded through their study that TTSS is an appropriate and feasible tool to predict the development of complications or mortality in mild trauma to the thorax [11]. TTSS was significant for predicting complications (area under the curve {AUC} = 0.848) and mortality (AUC = 0.856), with a cutoff value of eight points, having sensitivity and specificity of 66% and 94% to predict complications and 80% and 94% for predicting mortality, respectively [11].

Thoracic trauma takes a toll on the overall cost burden of the healthcare system, along with compromising the quality of life of the patients. The interventions employed in the management of thoracic trauma patients increase the overall morbidity of the patients, with postoperative pain, surgical site infections (SSIs), and hospital-acquired infections (HAIs) acting as the major contributory pillars [8]. Thereby, conservative management always bears an edge over interventional modalities on the grounds of SSIs, HAIs, and shorter lengths of hospital stay.

Limitations

In this study, only two modalities of management could be studied, and the outcomes of other modalities such as VATS and thoracotomy could not be studied. This was because no patients during the study duration required these interventions at our center. Also, CT-based volumetric analysis for lung tissue damage was not done in our study. Moreover, polytrauma patients were excluded from our study, due to the complex mechanism of interventions in them, which would not allow individual modality-wise analysis to be possible.

## Conclusions

According to our study results, the patients with hemothorax, sustaining blunt chest injury with ≤3 rib fractures, can be best managed by conservative means or by ICD, by keeping a close watch on the clinical condition of the patient. A TTSS of >7, across any number of rib fractures, was preferably predictive of management by ICD, while <7 value was favorable for conservative management. TTSS can be used as an important tool to predict the management modality in blunt chest injury patients with ≤3 rib fractures. However, for the generalization of the above results, similar studies with greater sample size, a broader spectrum of patients, and multicentric design and also those that include other treatment modalities such as video-assisted thoracoscopic surgery (VATS) and thoracotomy require to be conducted in the future. Nevertheless, this study and future studies on similar lines will have the implication of reducing the time taken to decide the mode of management by the use of the TTSS score and will also prevent unnecessary intervention by ICD, which has its own set of disadvantages for the patients.
